# Comparative energetics and kinetics of autotrophic lipid and starch metabolism in chlorophytic microalgae: implications for biomass and biofuel production

**DOI:** 10.1186/1754-6834-6-150

**Published:** 2013-10-19

**Authors:** Sowmya Subramanian, Amanda N Barry, Shayani Pieris, Richard T Sayre

**Affiliations:** 1 New Mexico Consortium, 100 Entrada Rd., Los Alamos, NM 87544, USA; 2 Bioscience Division, Los Alamos National Lab, M888, Los Alamos, NM 87545, USA; 3 Natural Sciences Division, Missouri Baptist University, One College Park Drive, St. Louis, MO 63141, USA

**Keywords:** Biofuel, Algae, Plant, Storage carbohydrate, Starch, Oil, Energetics, Photosynthesis, Biomass, Metabolism, Cultivation

## Abstract

Due to the growing need to provide alternatives to fossil fuels as efficiently, economically, and sustainably as possible there has been growing interest in improved biofuel production systems. Biofuels produced from microalgae are a particularly attractive option since microalgae have production potentials that exceed the best terrestrial crops by 2 to 10-fold. In addition, autotrophically grown microalgae can capture CO_2_ from point sources reducing direct atmospheric greenhouse gas emissions. The enhanced biomass production potential of algae is attributed in part to the fact that every cell is photosynthetic. Regardless, overall biological energy capture, conversion, and storage in microalgae are inefficient with less than 8% conversion of solar into chemical energy achieved. In this review, we examine the thermodynamic and kinetic constraints associated with the autotrophic conversion of inorganic carbon into storage carbohydrate and oil, the dominant energy storage products in Chlorophytic microalgae. We discuss how thermodynamic restrictions including the loss of fixed carbon during acetyl CoA synthesis reduce the efficiency of carbon accumulation in lipids. In addition, kinetic limitations, such as the coupling of proton to electron transfer during plastoquinone reduction and oxidation and the slow rates of CO_2_ fixation by Rubisco reduce photosynthetic efficiency. In some cases, these kinetic limitations have been overcome by massive increases in the numbers of effective catalytic sites, e.g. the high Rubisco levels (mM) in chloroplasts. But in other cases, including the slow rate of plastoquinol oxidation, there has been no compensatory increase in the abundance of catalytically limiting protein complexes. Significantly, we show that the energetic requirements for producing oil and starch relative to the recoverable energy stored in these molecules are very similar on a per carbon basis. Presently, the overall rates of starch and lipid synthesis in microalgae are very poorly characterized. Increased understanding of the kinetic constraints of lipid and starch synthesis, accumulation and turnover would facilitate the design of improved biomass production systems.

## Introduction

**Figure 1:**
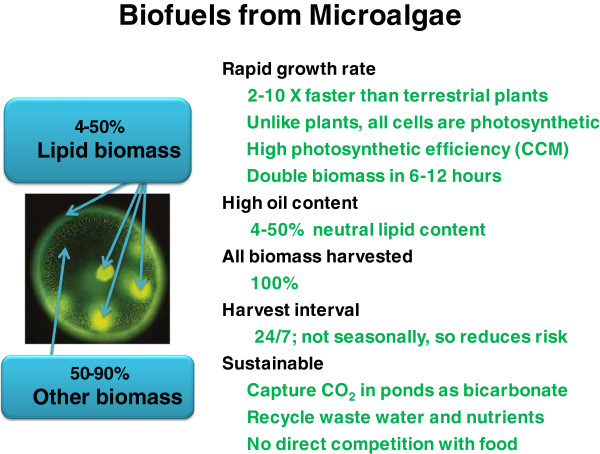
The advantages of algal biomass production systems. CCM; carbon concentrating mechanism.

Concerns about higher energy prices, finite fossil fuel reserves, rising atmospheric CO_2_ levels, and energy security have led to a growing interest in developing domestically produced renewable sources of energy using biomass production systems [1,2]. Given the low solar energy density on the earth’s surface and the inefficiencies of photosynthesis, robust biomass production systems that are optimized for the local environment and which have the greatest energy-return-on-investment are (EROI) desirable for producing biofuels. Recently, there has been growing recognition that microalgae have among the highest potential to produce the greatest biomass per unit area in the shortest period of time. Furthermore, microalgae have enhanced environmental sustainability characteristics since they can utilize inorganic carbon (bicarbonate) sequestered in ponds that can be captured from CO_2_ production point sources (power plants), unlike terrestrial plants. In addition, microalgae produce feedstocks (oils) for conversion to fuels that are compatible with the existing liquid transportation infrastructure [3]. Compared to crop plants, microalgae have 2–10 times the biomass yield potential. This enhanced productivity is in part attributed to the fact that all cells are photosynthetic unlike plants and that algae have active carbon concentrating mechanisms (CCMs) to enhance photosynthetic efficiency (Figure 1). Furthermore, some microalgae are capable of accumulating large amounts of lipids (up to 70% w/w) [4-7], can recycle water and nutrients from effluent streams, and do not directly compete with food production [2,8]. In addition, hydrocarbons produced by algae represent a potential means to store and sequester carbon [2]. Finally, from the energy security perspective a production failure in an algal pond can generally be brought back on line in a matter of days whereas a crop failure in a terrestrial crop production system may take up to a year before another harvest can occur.

Similar to all biomass production systems, however, microalgae have restricted cultivation areas. Cost-effective algal biomass production is constrained by topography (slopes less than 1%), water availability, temperature, nutrients (CO_2_, N and P), and the competing requirements for agricultural food production [5,9]. Production methods include open ponds, closed reactors, matrix-immobilized algae, and algal biofilms (for an in-depth review on production and harvesting techniques currently in use, see [10]). Several open pond systems have been evaluated at the pilot scale for microalgal growth, harvesting and biofuel production [11]. In contrast to open ponds, closed photobioreactors (PBRs) can be more productive on an aerial basis and allow stricter control over light, temperature, pH and nutrient inputs. However, recent comparative life cycle analyses of open pond versus closed PBR systems indicate that the higher capex and opex costs of closed PBRs make them prohibitively expensive to produce biofuels [12].

Unlike crop systems, many microalgae are also capable of heterotrophic growth and can use a variety of reduced carbon sources for biomass or oil accumulation. For example *Chlamydomonas reinhardtii* can assimilate acetate. Other species can be grown heterotrophically on sugars such as glucose which can enhance oil accumulation by as much as 900% [13]. Glycerol, a byproduct of the production of biodiesel production, can also be utilized for heterotrophic or mixotrophic growth by some microalgal species to achieve higher lipid productivity [14-16]. Although heterotrophic growth has been shown to produce high levels of desirable neutral lipids (for review, see [17]), addition of a reduced carbon source to algal ponds is not always feasible due to higher susceptibility to microbial contamination and cost. Finally, due to their low culture density (0.1% of mass) in ponds and small cell size (1–10 μm) considerable energy, accounting for up to 40% of production costs, may be required to harvest microalgae [8,18]. Substantial progress, however, is being made in the development of new technologies to reduce cultivation and harvesting costs (NAABB Consortium web site: http://www.naabb.org). Ultimately, for algal biofuel production systems to be financially, energetically and temporally successful, the financial- (FROI) and EROI for algal biofuel production systems must approach or be greater than that of competing fuel technologies. Economic analyses indicate that improvement of lipid content and growth rate are the predominant cost drivers for economical algal biofuel production [18].

In this review, we consider the kinetic and thermodynamic constraints of the cellular aspects of microalgal (Chlorophytic) biomass production systems. We demonstrate that the metabolic and physiological constraints impacting biomass accumulation are poorly characterized and need targeted research investment to facilitate the development of targeted strategies to improve biomass production and accumulation in microalgae. Much of our understanding of photosynthesis and carbon fixation stems from studies on cyanobacteria, Chlorophyta and higher plants. Because microalgae are a very diverse group of organisms in their entirety, we will restrict the bulk of our discussions to the Chlorophyta.

### Kinetics of biomass accumulation

Microalgae have a broad range of growth rates and energy densities depending on the species and growth conditions. Biomass productivity rates range from 15–30 g dry weight m^-2^ day^-1^[19,20] with an average energy content of 4.3 to 7.0 kcal/gdw [21,22]. The higher energy densities are typically associated with higher lipid contents. In general there is often a trade off, however, between cell division rates and the accumulation of energy storage products including storage carbohydrate (such as starch and glycogen) and neutral lipids or triacylglycerol (TAGs). Many studies have demonstrated that a variety of stress conditions that reduce growth rates lead to enhanced storage carbohydrate and/or oil accumulation [23-25]. Oil accumulation induced by stress may increase the energy content per dry weight by 50% or more but also may involve substantial lipid remodeling [24,25]. There are a number of factors, however, that determine overall rates of biomass accumulation at the cellular level. Some of these factors include: the absorption spectrum of the photosynthetic pigments, the optical cross-section of the light-harvesting apparatus, energy transfer efficiency, enzyme rate kinetics, the numbers and concentrations of energy transfer complexes (electron transfer chains) and enzyme catalytic sites, substrate concentrations, metabolite flux rates, metabolic compartmentalization and feedback control, respiration rates, and the partitioning of reduced carbon between new cell growth and division and energy storage [26-28].

### Kinetics of light capture and energy conversion

The earliest event in biological solar energy conversion is light capture. Energy capture and transfer by the light harvesting pigment-protein complexes is among the most energy efficient and fastest processes known in any biological system. Green algae (Chlorophyta) and higher plants initially capture photons via their light harvesting antenna complexes (LHC). The pigments associated with the LHC complexes in green algae account for about 80% of the total chlorophyll content with the remaining 20% of pigments associated with the proximal antenna and reaction center (RC) complexes where charge separation occurs [29]. In eukaryotic algae and plants the chlorophylls and carotenoids of the LHC complexes are bound to thylakoid membrane proteins in close association with the RCs. Energy transfer between pigments of the LHC complexes occurs on the femtosecond time scale [30]. Recent experimental and theoretical studies of electronic energy transfer processes in LHCs have revealed that energy transfer between pigments in the peripheral LHCs occurs at nearly 100% efficiency mediated by long-lived quantum coherence energy transfer processes [31-33]. Advances in techniques used to probe quantum coherence (2D- and multi-dimensional electronic spectroscopy) effects in energy transfer have provided a wealth of information on the mechanism and nature of energy transfer, charge separation and connectivity between the donor-acceptor states [32,34].

**Figure 2:**
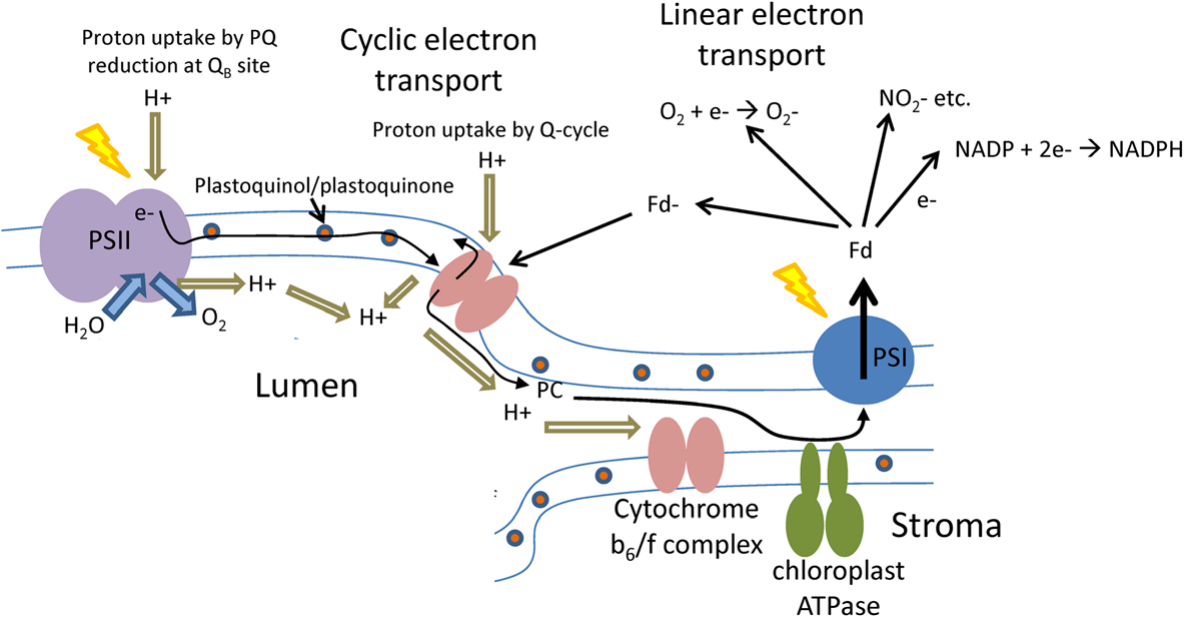
**Model of the photosynthetic electron transfer system of plants and Chlorophyta demonstrating the lateral membrane heterogeneity of the major protein complexes.** Abbreviations: Fd, ferredoxin; PC, plastocyanin; PQ, plastoquinone; PSI, photosystem I; PSII, photosystem II

The first kinetic constraints in energy transfer following light capture occur at the interfaces between the peripheral and proximal antenna as well as between the proximal antenna and the RC. For example, energy transfer between the proximal antenna complexes, CP43/CP47 and the Photosystem II (PSII) RC occurs at a time scale of 20–30 ps, while the subsequent primary charge separation in the PSII-RC occurs within 1–3 ps [29,35]. These kinetic bottlenecks result in some energy losses at higher (≥ 25% of full sunlight) than saturating light intensities. However, the greatest kinetic bottleneck in photosynthetic electron transfer is the coupled transfer of protons and electrons associated with the diffusion and oxidation of plastoquinol (PQH_2_) mediated by cytochrome b_6_/f (Cytb_6_f) complex [36,37]. This has significant implications for the efficiency of photon utilization at various light intensities. Further complicating the picture is the fact that each independent electron transfer complex in the photosynthetic apparatus has substantially different rate-limiting kinetics. The overall rate-limiting steps for charge separation or electron transfer in the photosystem I (PS I), PS II and cytb_6_f complex are 1 ns, 1 μs and 10 ms, respectively (Figure 2) [38-40]. Additional proton coupled steps also kinetically constrain energy transduction including the slow diffusion rates of plastoquinone [37] as well as the relatively slow turnover (100–200s^-1^) of the ATP synthase complex [41,42]. It is noteworthy that a variety of metabolic control mechanisms may also impact rate kinetics. For example, the ATP synthase can function in the reverse direction *viz.*, hydrolysis of ATP depending on the trans-thylakoidal pH as well as its redox control by thioredoxins [43] leading to reduction in ATP synthesis rates. However, in a recent paper by Yamori *et al*., in which the authors describe the effects of reduced ATP synthase and Cytb_6_f contents on overall electron transfer rates and CO_2_ assimilation rates, they observed that reductions in Cytb_6_f content had the greatest impact on overall electron transfer rates, and not alterations in ATP synthase levels [44]. To further complicate matters the activity of the ATP synthase complex has been shown to be modulated by CO_2_ concentrations [43,45-47].

Significantly, rate-limiting steps in electron transfer indirectly impact the means by which the energy of chlorophyll excited states in the LHC complex are dissipated either by photochemistry or by re-emission of trapped energy as heat or fluorescence. The relative proportion of captured energy that is converted into charge separated states or dissipated by non-photochemical quenching (NPQ) mechanisms is determined by both the light intensity, the optical cross-section of the light harvesting apparatus and the kinetics of electron transfer processes. In *Chlamydomonas* the apparent optical cross section of the photosystem II (PS II) antenna complex is about 70 Å^2^[48]. At a photon flux density (400–700 nm) of 2,000 μmole photons/m^2^/sec, equivalent to full sunlight intensity, the rate of photon capture approaches 1.2 photons per ms per PSII complex. This rate of photon capture is five to eight times faster than the rate-limiting step (PQH_2_ oxidation) in photosynthetic electron transfer (discussed below). Thus, at full sunlight intensity about 75% of the energy that is captured by the LHC antenna complex does not drive photochemistry but is dissipated as heat or fluorescence by non-photochemical quenching (NPQ) mechanisms [49,50]. Similar inefficiencies in light capture and conversion have been observed in whole plants. Björkman *et al.*, [51] determined from a study of 42 species of plants that the average photochemical quantum yield for oxygen evolution was 0.108. Similarly, Long *et al.*, [52] measured a maximum, average photochemical quantum yield of 0.093 from CO_2_ exchange measurements from 11 species of C3 plants. These studies indicate that most plants and algae have light harvesting systems that are larger than necessary for efficient light capture and energy conversion. Consistent with this observation, it has been demonstrated that reductions in the size of the LHC complex associated with the inhibition of chlorophyll b synthesis in green algae leads to higher rates of photosynthesis in algal cultures at high light intensities [33,50,53,54]. Recent studies using algae engineered to have a range of peripheral light-harvesting antenna sizes have demonstrated that the optimal antenna size for efficient conversion of light into chemical energy in green plants and in Chlamydomonas mutants is approximately 30% smaller than wild type, a size that is consistent with efficient coupling of the rate kinetics of light capture with the limitations of downstream electron transfer steps [33].

### Kinetics of carbon fixation

**Figure 3:**
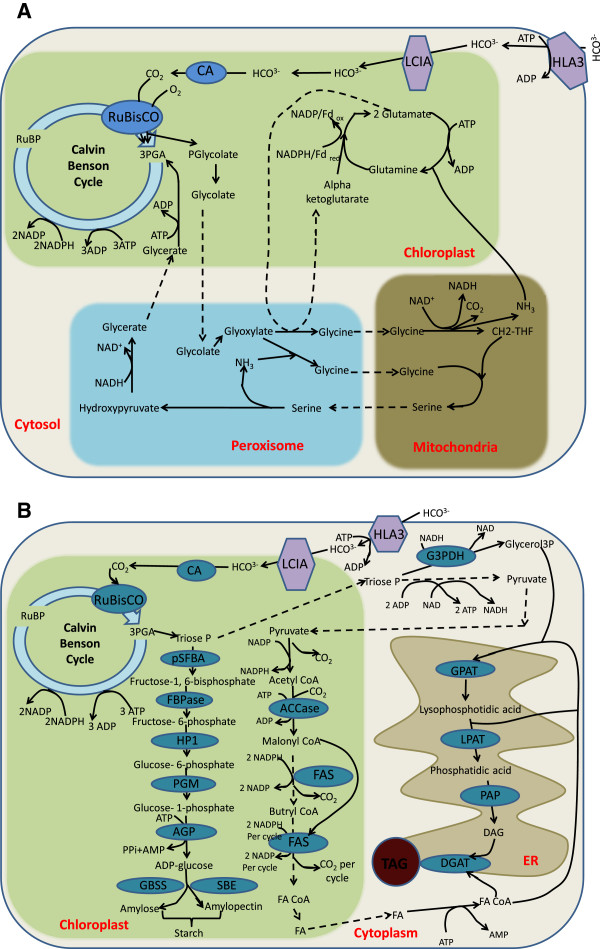
**Metabolic pathways for lipid and carbohydrate synthesis. A.** Outline of the Calvin-Benson and photorespiration cycles. **B.** Description of storage carbohydrate and TAG synthesis. See list of abbreviations for enzyme names.

**Figure 4:**
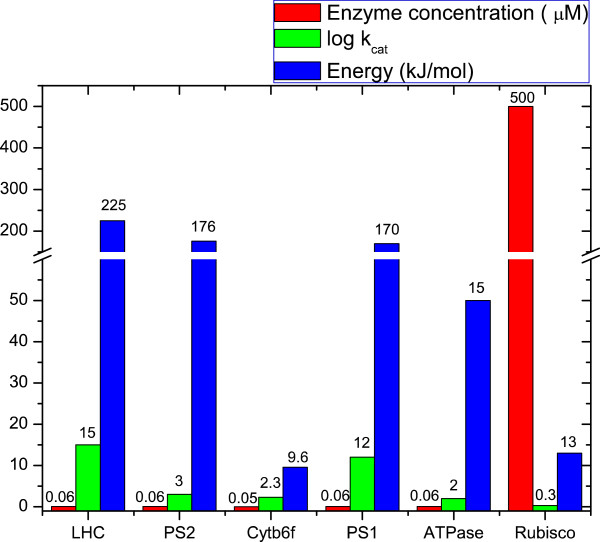
**Graphical representation of the free energy change, enzyme complex concentration and catalytic turnover numbers (k**_**cat**_**) of various components of the photosynthetic apparatus.** Data for the plot were obtained from [38-40].

The overall rate-limiting step in photosynthesis, however, is the initial fixation of CO_2_ by the enzyme Rubisco. Rubisco has a turnover rate of 2–10 molecules of CO_2_ s^-1^, and is one of the slower enzymes in nature [55]. To compensate for the slow rate of CO_2_ fixation by Rubisco, plants and algae have very high concentrations of Rubisco active sites, on the order of 5 mM or 1,000 times the number of PSII and PSI RCs (Figures 3 and 4). Due to the very large number of total Rubisco catalytic sites the overall rates of carbon dioxide fixation approach maximum rates of electron transfer. The efficiency of Rubisco is reduced, however, by the competitive fixation of oxygen versus carbon dioxide and the fact that much of the enzyme may be in an inactive state. Rubisco oxygenase activity reduces the efficiency of CO_2_ fixation, the regeneration of the substrate RuBP, and results in the loss of previously fixed carbon during the decarboxylation of serine in the photorespiratory pathway (Figure 3) [56]. The net reduction in CO_2_ fixation by the photorespiratory pathway has been estimated to be 25% of the maximum carboxylation efficiency [56]. Many microalgae facultatively (when grown in air) reduce photorespiratory losses by actively pumping bicarbonate into the cells where it is converted back to CO_2._ This can occur in specialized sub-organellar structures known as pyrenoids in eukaryotic algae where carbonic anhydrase is closely associated with Rubisco to facilitate the conversion of bicarbonate to carbon dioxide [57]. This type of algal CCM is best characterized in the green alga, *Chlamydomonas reinhardtii*[57,58]. In *Chlamydomonas*, bicarbonate is actively transported across the plasmamembrane by an ABC-type transporter (HLA3) that presumably requires at least one additional ATP/CO_2_ fixed, as shown by gene knockout studies [59]. This additional ATP demand to pump carbon dioxide into the cell is met by cyclic photophosphorylation [60]. Subsequently, bicarbonate is transported into the plastid by the LCIA transporter and dehydrated by carbonic anhydrase to generate CO_2_. Similarly, cyanobacteria employ a number of bicarbonate transporters and carbonic anhydrases to increase the CO_2_ levels in specialized compartment called carboxysomes, where carbon fixation occurs. In diatoms, CCM occurs by both actively increasing CO_2_ levels near the site of carbon fixation (Rubisco), and C4 fixation.

It has been demonstrated in plants that there are additional kinetic constraints in the Calvin-Benson Cycle associated with the limiting abundance of enzymes that have very high equilibrium constants including; Seduheptulose bisphosphatase (SBPase), fructose bisphosphatase (FBPase) and aldolase [38]. Overexpresison of the enzymes, FBPase and SBPase in tobacco lead to increased sugar and storage carbohydrate content, improving biomass yield [61]. Similar results have been achieved in rapidly dividing algal cells (Sayre, unpublished results). Metabolic flux rates through the Calvin-Benson Cycle have been described by a number of labs [62,63], however carbon flux through carbon storage and sequestration pathways leading to storage carbohydrate and oil synthesis have not been well characterized in microalgae. Thus, it is not possible to account for potential differences in rate kinetics for overall storage carbohydrate and oil synthesis.

### Thermodynamics of energy capture and conversion in algae

The thermodynamic efficiency of light capture and energy conversion by the photosynthetic electron transfer system as well as the efficiency of carbon fixation, metabolism, respiration and carbon sequestration determine the overall energy efficiency of solar energy conversion into chemical energy present in biomass [64,65]. Of the total solar radiation that reaches the earth’s surface, photosynthetically active radiation (PAR) is limited to 400–700 nm for most eukaryotes, and accounts for less than 50 % of the total solar photon flux [64].

In plants and the Chlorophytes, the light harvesting complexes (LHCs) or antenna protein-pigment complexes are comprised of a superfamily of proteins. The LHC proteins bind chlorophylls and carotenoids (Car) that capture light and transfer energy to the reaction centers (RCs). Chlorophyll *a* (Chl *a*), is present in all eukaryotic photosynthetic organisms. Additional species of chlorophyll including Chl *b*, Chl *c* or Chl *d* along with carotenoids (Car) expand the light absorption spectrum of the light harvesting antenna complexes [66]. In green algae (Chlorophyta) and higher plants, the peripheral antenna include Chl a/b-binding proteins whose pigments absorb predominantly in the blue (430–455 nm) and red (650–690 nm) region of the solar spectra.

In plants and green algae, the LHC proteins associated with photosystem II (LHCII/peripheral antenna) form a trimeric complex (major LHC) in association with three other proteins (minor LHC). Typically, the PSII RC and proximal antenna proteins of green algae and higher plants bind about 6 Chl *a*/2Car and 28 Chl *a,* respectively [67,68]. The PSII crystal structure of spinach demonstrates that the major [67,68] LHCII binds about 8 Chl *a*/6 Chl *b*/4 Car per monomer [69], while the minor LHCII bind about 5–6 Chl *a*/2-5 Chl *b*/2 Car. Structural studies of PSI revealed that the PSI associated LHC (LHCI) is a tetrameric protein with 14 Chl *a*+*b* molecules bound per monomer [70], and the PSI core complex binds about 200 molecules of Chl *a* and about 20 carotenoids.

Members of the LHC family also bind carotenoids [71]. The carotenoids serve two roles in the LHC: 1) They expand the spectra of light captured by the photosystems, and 2) dissipate the excess excitation energy from Chl through non-photochemical quenching (NPQ). As previously discussed, under high light conditions electron transfer is saturated, and this results in the accumulation of Chl singlet excited states (^1^Chl*). When photochemistry is saturated, ^1^Chl* relax to the ground state by emitting energy as heat and/or fluorescence or by singlet-singlet exciton annihilation. During the latter process, spin inversion results in the formation of ^3^Chl* which can interact with ^3^O_2_ to produce singlet ^1^O_2_ species, a very potent oxidant that causes damage to the photosynthetic apparatus or photoinhibition [72-74]. In addition, excited state Chls can be quenched by zeaxanthin [75] resulting in the emission of heat. This process is induced by increased acidification of the lumen, which triggers the xanthophyll cycle, wherein de-epoxidases convert violaxnathin to zeaxanthin. However, the exact mechanism by which zeaxanthin quenches the excess energy from Chl excited states remains a subject of controversy [49,76,77].

### Thermodynamics of carbon reduction

The quantum efficiency of primary charge separation in photosystem I and II is quite high. Energy transfer from the PSII antenna pigments to the RC primary electron donor chlorophyll (P680) in PSII leads to charge transfer. The primary electron acceptor, pheophytin (Phe) accepts an electron from P680* to form a radical pair state (P680^+^Phe^-^). The radical pair in turn reduces the secondary electron acceptor Q_A_, a single electron-accepting plastoquinone molecule, with lower energy than Phe^-^ resulting in the stabilization of the charge separated state. The transfer of electrons in an energetically downhill fashion contribute to the thermodynamic efficiency and the high quantum efficiency (0.8-0.9) of the charge separation process [78,79]. A similar process of charge separation and thermodynamically down-hill energy transfer occurs in PSI-RC (P700). The photoexcited P700* reduces the primary acceptor Chl molecules (A_0_ & A_1_) which stabilizes primary charge separation with a quantum efficiency of ~ 1 [47,80].

The overall apparent quantum or thermodynamic efficiency of photosynthesis is generally normalized, however, to the number of photons required to evolve one molecule of O_2_ or fix one molecule of CO_2_ (Figure 2 and 3). A theoretical maximum quantum efficiency of 8 red (680 nm) photons/CO_2_ fixed was proposed by Emerson *et al.*, in the late 1950s [81]. Evidence based on the Emerson enhancement effect, which identified the role of the two photosystems working in series, and determination of the quantum requirement through calorimetric studies by Arnold *et al*., [82] supported a quantum requirement of 10–12 photons per CO_2_ fixed. While, the difference in the quantum yield owing to the difference in the measurement technique (O_2_ evolution vs. CO_2_ assimilation) can be appreciated, recent reports indicate that the quantum requirement for CO_2_ fixation in plants in the absence and presence of photorespiration is ~9 and ~ 10 photons, respectively [83,84]. However, the experimental photosynthetic quantum efficiency ranges as high as 20 for plants under stress [85]. Given that the stoichiometric ratio of ATP and NADPH per CO_2_ fixed is 3:2 (from Calvin cycle) and that 4 reducing equivalents are required to generate 2 NADPH or one O_2,_ the magnitude of the proton motive force necessary to drive synthesis of sufficient ATP to fix CO_2_ then becomes the critical factor to balance the energy requirements for CO_2_ fixation. Recent experimental evidence suggests that the H^+^/ATP ratio is approximately 4 [86-88]. These results are consistent with structural inferences from the ATP synthase complex. The CF_0_ rotor ring of the CF_0_-CF_1_ complex of the ATP synthase has 14 C-subunits, each of which must be sequentially protonated to drive ATP synthesis. The fact that the ATPase has 3 catalytic sites that must complete a rotation cycle to generate 3 ATPs, indicates that the H^+^/ATP ratio is approximately 4.67 or 14 protons/3ATP [89,90]. Since the proton requirement/ATP synthesized is 4 and 4 ATPs are required for each CO_2_ pumped (1 ATP) and fixed (3 ATP) the proton requirement (16 H^+^) to produce sufficient ATP to fix CO_2_ cannot be met by linear electron transfer of 4 electrons to produce 2 NADPH. Additional proton motive force can be generated by a variety of mechanisms. On average, each light-driven charge separated state generates the stoichiometric equivalent of 1.25 protons/electron. This occurs via a combination of multiple proton generating or transducing steps including water splitting (1 H^+^/e-), PQH_2_ oxidation (1 H^+^/e-), proton transfer via the Q cycle (1.5 H^+^/e-), and cyclic photophosphorylation (1–1.5 H^+^/e-). Considering water splitting and the Q-cycle alone, twelve proton equivalents are expected to be generated from 8 photochemical events associated with the production of 2 NADPH molecules [82]. Since the proton requirement/ATP synthesized is > 4 and 4 ATPs are required for each CO_2_ pumped and reduced by the Calvin-Benson Cycle the proton requirement (~16 H^+^) to produce sufficient ATP to fix CO_2_ cannot be met by linear electron transfer. The additional proton requirement must be provided by cyclic photophosphorylation [91]. In cyclic photophosphorylation, electrons derived from ferredoxin reduce plastoquinone in the cytochrome b_6_f complex. Reduced ferredoxin is generated by photosystem I. Reduced PQ molecules are protonated on the stromal side (Q_I_ site) of the Cytb_6_f complex and are re-oxidized at the Q_O_ site on the lumenal side of the thylakoid, thus increasing the proton gradient at the expense of NADPH synthesis.

As stated previously, cyclic photophosphorylation is also essential for active bicarbonate uptake in green microalgae [92,93]. In air-grown chlorophytic microalgae such as *Chlamydomonas* the biosynthesis of any reduced carbon storage product begins with the active uptake of bicarbonate and the subsequent reduction of the imported inorganic carbon or CO_2_ via the Calvin-Benson Cycle. For *Chlamydomonas*, one additional ATP is required to support its active transport into the cell by a plasmamembrane localized ABC-type bicarbonate transporter, the HLA3 protein (David Kramer, personal communication). Recent evidence indicates that bicarbonate uptake by the HLA3 transporter requires one additional ATP/CO_2_ fixed. Bicarbonate is then transported into the chloroplast stroma by the LCIA transporter and eventually to the thylakoid lumen by another putative bicarbonate transporter. Carbonic anhydrases localized in the stroma and the lumen, catalyze the conversion of bicarbonate to CO_2_, effectively elevating the internal CO_2_ concentration by 10-fold and competitively inhibiting the oxygenase reaction of Rubisco [57]. This inorganic carbon pumping system effectively reduces photorespiration by more than 90% improving overall photosynthetic efficiency [94,95].

### Storage carbohydrate (starch) and oil production during autotrophic growth

During active photoautotrophic growth carbon in excess of that required for metabolism, respiration and growth is stored typically as carbohydrate or TAGs. As shown in Table 1, the storage carbohydrate (polysaccharides) or oil (lipid) content of algae can range anywhere from 6 to 64% of the total biomass. Typically, only carbohydrates and oils are utilized for the production of commercial fuels, however. For this reason, as well as to simplify the thermodynamic considerations for biofuels production, we only consider the energetic costs for carbohydrate and oil production. These calculations are made under standard physical conditions since we do not have a comprehensive understanding of the steady state pool size of products and reactants typically found in cells (Table 2). For stoichiometric considerations, we compare the energetic requirements for the synthesis of 6 TAG molecules containing fatty acids with the following chain lengths and degree of saturation, C16:0, C18:1, and C18:3, and having the molecular formula C_55_H_98_O_6_. We compare these energetic requirements to the energetic required to synthesize starch (55 glucose) having the same number of carbon atoms (330) as 6 TAGs. We begin the energy accounting with the active import of inorganic carbon by HLA3 followed by the synthesis of triose-phosphate produced in the Calvin-Benson Cycle. Triose-phosphate is the substrate for fatty acid and TAG synthesis as well as glucose and starch synthesis.

**Table 1:** Protein, carbohydrate and lipid fractions in several algal species

**Algal species**	**Protein**	**Carbohydrates**	**Lipids**
*Anabaena cylindrica*[96]	43–56	25–30	4–7
*Aphanizomenon flos-aquae*[96]	62	23	3
*Chlamydomonas reinhardtii*[96]	48	17	21
*Chlorella minutissima*[21]	9–24	14–42	31–57
*Auxenochlorella protothecoides*[97]	11–26	17–24	25–54
*Auxenochlorella protothecoides*[21]	36–38	41–52	11–23
*Chlorella pyrenoidosa*[96]	57	26	2
*Chlorella emersonii*[21]	28–32	11–41	29–63
*Chlorella sorokiniana*[21]	42–45	32–38	20–22
*Chlorella vulgaris*[96]	51–58	12–17	14–22
*Chlorella vulgaris*[21]	7–29	51–55	18–40
*Dunaliella salina*[96]	57	32	6
*Euglena gracilis*[96]	39–61	14–18	14–20
*Nannochloropsis* sp. [98]	23–59	5–17	9–62
*Porphyridium cruentum*[96]	28–39	40–57	9–14
*Scenedesmus obliquus*[96]	50–56	10–17	12–14
*Spirogyra* sp. [96]	6–20	33–64	11–21
*Arthrospira maxima*[96]	60–71	13–16	6–7
*Spirulina platensis*[96]	46–63	8–14	4–9
*Synechococcus* sp. [96]	63	15	11

Presented as a percentage of total dry weight. Related references are given in brackets.

**Table 2:** Energetic (ATP and NAD(P)H) requirements for the synthesis of TAG and storage carbohydrate per unit carbon

	**ATP/NAD(P)H**
**TAG synthesis partial reactions:**	
3 CO_2_ → 3-phosphoglyceraldehyde→ acetylCoA	12 ATP, 4 NAD(P)H + CO_2_
AcetylCoA_(n-1used)_ mediated fatty acid elongation (e.g., C16:0)	7 ATP + 14 NADH
Fatty acid desaturation	1 NADH equivalent/bond desaturated
3 CO_2_ → Glycerol	9 ATP + 7 NAD(P)H
**TAG (C**_**55**_**H**_**98**_**O**_**6**_**) synthesis summary:**	
26 acetyl CoA =	312 ATP, 104 NAD(P)H
Fatty acid elongation C16:0 + 2 C18:0 =	23 ATP, 46 NAD(P)H
4 desaturations	4 NAD(P)H equivalents
1 glycerol	9 ATP, 7 NAD(P)H
**Total**	344 ATP, 160 NAD(P)H
**Per carbon**	6.25 ATP, 2.93 NAD(P)H
**Starch /glycogen synthesis partial reactions:**	
6 CO_2_ → 2 3-phosphoglyceraldehyde → glucose	24 ATP, 12 NAD(P)H
Glucose → storage carbohydrate	1 ATP/glucose
**Starch synthesis (55 units, 330 C) summary:**	
**Total for 55 glucose**	1375 ATP, 660 NAD(P)H
**Per Carbon**	4.16 ATP, 2 NAD(P)H

Overall, the synthesis of a typical TAG (C16:0, C18:1 and C18:3; C_55_H_98_O_6_) includes the energy required for acetyl CoA synthesis, fatty acid synthesis, fatty acid desaturation, and glycerol synthesis. As summarized in Table 2, TAG (C_55_H_98_O_6_) synthesis requires 6.3 ATP/carbon and 2.9 NAD(P)H/carbon.

Glucose is derived from the condensation of two molecules of 3-phosphoglyceraldehyde. Activation of glucose units for starch polymerization requires the consumption of one additional ATP for the synthesis of ADP glucose. Overall, however, the energy required for storage carbohydrate synthesis is dominated by the energy required for carbon reduction by the Calvin-Benson Cycle. In total, the energy required for starch synthesis is 4.2 ATP/carbon and 2 NAD(P)H/carbon. Thus on a per carbon basis, the ATP and NAD(P)H requirements for starch synthesis are 50% and 45% lower, respectively than those required for TAG synthesis. If we assume the free energy of ATP hydrolysis and NADH oxidation (with molecular oxygen) are -50kJ/mole and -220 kJ/mole, respectively, then the energy cost per carbon for TAG synthesis (993 kJ/C) is 53% greater than for storage carbohydrate (650 kJ/C) synthesis under standard conditions. These energetic values are compromised, however, by limited knowledge of the *in vivo* concentrations of substrates and products and the energy of hydration.

Cells metabolize lipids and carbohydrates both for carbon skeletons to build other molecules as well as for energy metabolism. The efficiency of energy recovery from lipid and storage carbohydrate metabolism has been well studied in plants and animals. When saturated fatty acids are oxidized via the β-oxidation pathway and the citric acid cycle, the energy recovery is approximately 6.6 ATP equivalents/C. For glucose oxidation via glycolysis and the citric acid cycle the energy recovery is 5 ATP equivalents/carbon [99]. Thus, the energy recovery from fatty acid (lipid) oxidation is 32% greater than glucose (storage carbohydrate) oxidation. Similar results are obtained if we directly combust TAGs or storage carbohydrate to produce energy (Table 3). Since TAGs have an average energy density (heat of combustion) of 38 kJ/g and storage carbohydrate has an average energy density of 15.5 kJ/g, then for an equivalent number of carbons, the energy content per carbon of TAG is 41% greater than that for starch (Table 3).

**Table 3:** Relative energy content (heat of combustion)/carbon for TAG and starch

	**Energy**
Mass for an equivalent number of moles of carbon (330) in TAG and starch	
6 moles TAG (C_55_H_98_O_6_) = 5124 g	
55 moles glucose in starch (C_6_H_12_O_6_, monomer) = 8928 g	
Energy content of an equivalent number of moles of carbon (330) in TAG and starch	
330 C = 5124 g TAG x 38 kJ/g TAG = 194,712 kJ/330C	= 590 kJ/C
330 C = 8928 g starch x 15.5 kJ/g storage carbohydrate = 138,384 kJ/330C	= 419 kJ/C

Interestingly, the relative biological energy input/carbon for TAG synthesis versus starch synthesis is substantially greater (53%) than the relative energy return from TAGs versus starch obtained either from respiration (32% greater for TAG than storage carbohydrate) or from direct combustion (41% greater for TAG than storage carbohydrate). Much of the increased energy costs for TAG versus storage carbohydrate synthesis can be attributed to the loss of reduced carbon that occurs during the conversion of pyruvate to acetyl CoA. Twenty six reduced carbon equivalents are lost during the decarboxylation of sufficient pyruvate to synthesize a typical TAG (C_55_H_98_O_6_) molecule. Overall, these results beg the question: Does it make sense to engineer algae with enhanced oil levels versus engineering algae with high carbohydrate levels if the EROI for TAGs is 10-20% lower than that for starch? If the biofuel feedstock is to be used at a refinery to make diesel, Jet Propellant 8 (jet fuel JP8) or gasoline, however, then oil has a lower downstream energy cost to produce reduced fuels than does glucose due to reduced hydrogen requirements.

### Biological implications of storage carbohydrate and oil accumulation in microalgae

What is missing from this discourse on the energetics and kinetics of starch and oil accumulation in algae is a greater understanding of overall cellular metabolism, flux rates, and cellular compartmentalization. Parameters that can impact the yields of starch and oil metabolism include; the kinetics, levels, and allosteric regulation (including feedback control) of the enzymes involved in their biosynthesis as well as the channeling of substrates and products through these and competing pathways. For example, starch accumulation in plastids can exert a feedback inhibition on photosynthesis, and so adequate sink strength is often necessary to achieve the greatest impact from improvements in photosynthetic rates [79,100,101]. Similarly, it has recently been shown that the substrates for TAG synthesis, acyl CoAs, can regulate the early dedicated events in fatty acid synthesis including acetyl CoA carboxylase activity [102]. There is also the role that these energy storage molecules play as substrates for the synthesis of other biologically important molecules.

### Summary

It is argued that kinetic and thermodynamic constraints that determine the biomass or energy yield of biological systems are currently very poorly characterized. We have described kinetic constraints within the photosynthetic electron transfer apparatus and Calvin-Benson cycle that impact the efficiency of light utilization. Some of these constraints include; the coupling of proton to electron transfer associated with plastoquinone reduction and oxidation, the slow turnover numbers of Rubisco, and limiting concentrations of enzymes or effective enzyme catalytic turnover numbers. In some cases, these constraints can be overcome by increases in the numbers of catalytic sites or enzyme concentrations. In other cases, evolution has not provided an apparent resolution to the slow kinetics. This opens opportunities for metabolic engineering strategies to increase photosynthetic efficiency. For example, increasing the plastoquinone pool size could buffer transients in fast PSII and slow Cytb_6_f electron transfer rates. Alternatively, increasing the activity or levels of the cytochrome b_6_f complexes may accelerate electron transfer rates. In addition, the importance of optimizing Rubisco efficiency by concentrating CO_2_ at the active site requires greater cyclic photophosphorylation activity to produce sufficient ATP for active bicarbonate uptake.

Lastly, by comparing the energy requirements for TAG and starch synthesis, we find that storage carbohydrates such as starch are energetically cheaper to make than TAG on a per carbon basis. This differential energy cost is largely attributed to the loss of fixed carbon occurring during the decarboxylation of pyruvate to synthesize acetyl CoA, an obvious target for metabolic engineering. Moreover, the higher energy density of TAG does not compensate for the energy investment. Thus, storage carbohydrate accumulation may be an effective strategy for efficient chemical energy accumulation in algae.

## Abbreviations

ACCase: Acetyl CoA carboxylase; AGP: Adenosyl glucose phophotransferase; CA: Carbonic anhydrase; CCM: Carbon concentrating mechanism; DGAT: Diacylglycerol acyltransferase; DOE: US Department of Energy; EROI: Energy return on investment; FAS: Fatty acid synthase complex; Fd: Ferredoxin; FBPase: Fructose bisphosphatase; FROI: Financial return on investment; G3PDH: Glycerol-3-phosphate dehydrogenase; GPAT: Glycerol-3-phosphate acyltransferase; GBSS: Granular bound starch synthase; HPI: Hexose phosphate isomerase; JP8: Jet propellant 8 jet fuel; LHC: Light harvesting complex; LPAT: Lysophosphatidate acyltransferase; NAABB: National Alliance for Advanced Biofuels and Bioproducts (DOE algal biomass program); PAP: Phosphatidic acid phosphatase; PAR: Photosynthetically active radiation (400–700 nm); PC: Plastocyanin; PQ: Plastoquinone; PSI: Photosystem I; PSII: Photosystem II; pSFBA: Plastidial Sedulose/Fructose-Bisphosphatase Aldolase; RC: Reaction centers of photosystem I or II; SBE: Starch branching enzyme; TAG: Triacylglycerol.

## Competing interests

Dr. Richard Sayre is Chief Technology Officer for Phycal Inc.

## Authors’ contributions

All authors contributed equally to this publication. More specifically, SS contributed to writing sections on the energetics and kinetics of photosynthesis, organizing references and Figure 4. AB contributed to writing the introduction and summary, organizing the draft and references and Table 1 and Figure 2. SP contributed to writing on storage carbohydrate and oil metabolism and Figure 3. RS contributed to overall organization and thematic focus, energy calculations, Tables 2 and 3, Figure 1, and final editing. All authors read and approved the final manuscript.

## Authors’ information

Dr. Sowmya Subramanian is an associate research scientist at the New Mexico Consortium working on the metabolic engineering of electron transfer processes and carbon fixation to enhance photosynthetic efficiency.

Dr. Amanda N. Barry is a postdoc at LANL working on engineering algal bioflocculation and assessment of algal biomass productivity using controlled photobioreactors.

Dr. Shayani Pieris is an algal biotechnologist formerly at the New Mexico Consortium working on the enhancing of oil accumulation and photosynthesis in transgenic algae.

Dr. Richard Sayre has a joint appointment with the New Mexico Consortium and Los Alamos National Laboratory and is Senior Research Scientist and Scientific Director for the Center for Advanced Biofuel Systems and the National Alliance for Advanced Biofuels and Bioproducts.
